# Quantification of a Glucocorticoid Profile in Non-pooled Samples Is Pivotal in Stress Research Across Vertebrates

**DOI:** 10.3389/fendo.2018.00635

**Published:** 2018-10-23

**Authors:** Johan Aerts

**Affiliations:** ^1^Stress Physiology Research Group, Faculty of Pharmaceutical Sciences, Ghent University, Ostend, Belgium; ^2^Stress Physiology Research Group, Animal Sciences Unit, Flanders Research Institute for Agriculture, Fisheries and Food, Ostend, Belgium

**Keywords:** vertebrate, stress, HPI, HPA, glucocorticoid, profile

## Abstract

Vertebrates are faced continuously with a variety of potential stressful stimuli and react by a highly conserved endocrine stress response. An immediate catecholamine mediated response increases plasma glucose levels in order to prepare the organism for the “fight or flight” reaction. In addition, in a matter of minutes after this (nor)adrenaline release, glucocorticoids, in particular cortisol or corticosterone depending on the species, are released through activation of the hypothalamic-pituitary-interrenal (HPI) axis in fish or hypothalamic-pituitary-adrenal (HPA) axis in other vertebrates. These plasma glucocorticoids are well documented and widely used as biomarker for stress across vertebrates. In order to study the role of glucocorticoids in acute and chronic stress and gain in-depth insight in the stress axis (re)activity across vertebrates, it is pivotal to pin-point the involved molecules, to understand the mechanisms of how the latter are synthesized, regulated and excreted, and to grasp their actions on a plethora of biological processes. Furthermore, in-depth knowledge on the characteristics of the tissues as well as on the analytical methodologies available for glucocorticoid quantification is needed. This manuscript is to be situated in the multi-disciplinary research topic of glucocorticoid action across vertebrates which is linked to a wide range of research domains including but not limited to biochemistry, ecology, endocrinology, ethology, histology, immunology, morphology, physiology, and toxicology, and provides a solid base for all interested in stress, in particular glucocorticoid, related research. In this framework, internationally validated confirmation methods for quantification of a glucocorticoid profile comprising: (i) the dominant hormone; (ii) its direct precursors; (iii) its endogenously present phase I metabolites; and (iv) the most abundant more polar excreted exogenous phase I metabolites in non-pooled samples are pivotal.

## Key concepts

### Accurate identification and quantification of stressors experienced by an individual

The sheer diversity in potential stressors, individual perception and subsequent reaction to these stressors, and the plethora of metabolic processes mediated by glucocorticoids render accurate identification and quantification of the stressors experienced by an individual pivotal.

### Analysis of the dominant glucocorticoid is affected by other steroids

Glucocorticoid quantification can be biased by (i) the less dominant hormone; (ii) other steroids; (iii) direct precursors of the dominant hormone and the dominant hormone itself produced in extra-interrenal or extra-adrenal tissues; (iv) phase I metabolites present in the body; and (v) phase I metabolites present on the sample as contaminants.

### Analysis of the dominant glucocorticoid is affected by the sample tissue

Results can be enhanced or suppressed by tissue specific compounds, and potential effects should be analytically validated.

### Analysis of the dominant glucocorticoid is affected by the analytical methodology used

Glucocorticoid analysis should best be performed using confirmation methods. Hereby, UPLC-MS/MS is considered the gold standard for quantitation of glucocorticoids in complex biological tissues as it has the needed sensitivity, selectivity and the advantage of having the capability to perform multi-analyte assays, even across compound classes.

### Analysis of the dominant glucocorticoid is affected by the lack of analytical validation

Methods should best be developed in an EN ISO/IEC 17025 regulated environment and analytically validated according the criteria of international standards to ensure full traceability and quality of the results in time.

## Introduction

Moberg (1) defined stress as “a highly complex multi-dimensional phenomenon promoted by several noxious or unpredictable stimuli (stressors) that cause a physiological response (stress) aimed to maintain or recover the body homeostasis.” Stressors are diverse and generally classified based on their: (i) type (i.e., chemical, physical, and psychological); (ii) duration [i.e., transitory (acute) or long-term (chronic)]; (iii) severity; (iv) (un)predictability; and (v) (un)controllability (2). Hereby, stress can be perceived as harmful or negative (distress), as well as a neutral or even as a positive condition (eustress) (3).

Organisms are faced continously with a variety of potential stressful stimuli and have developed over time a plethora of mechanisms to cope with changes and challenges in their environment (4). When faced with such stressful stimuli, vertebrates, ranging from fish to humans, react by a highly conserved endocrine stress response. An immediate catecholamine mediated response increases plasma glucose levels in order to prepare the organism for the “fight or flight” reaction (5). In addition, in a matter of minutes after this (nor)epinephrine [(nor)adrenaline] release, glucocorticoids, in particular cortisol (11β,17α,21-trihydroxypregn-4-ene-3,20-dione or C_21_H_30_O_5_) or corticosterone (11β,21-dihydroxypregn-4-ene-3,20-dione or C_21_H_30_O_4_) depending on the species, are released through activation of the hypothalamic-pituitary-interrenal (HPI) axis in fish (6) or hypothalamic-pituitary-adrenal (HPA) axis in other vertebrates (2). These plasma glucocorticoids are widely used as biomarker for stress across vertebrates (7, 8) and considered as adaptation hormones as they mediate a redistribution of energy (i.e., glucose) in order to restore pre-stress conditions. However, failure to regain homeostasis (maladaptation) will inevitably lead to chronic stress making the individual prone to the detrimental effects of glucocorticoid mediated actions (e.g., decreased growth, decreased reproduction, immune suppression, increased mortality). In the concept of “allostasis” [i.e., constancy through change by resetting the set-points for homeostasis in accordance to environmental cues (9, 10)], this situation can be described as: the transition from allostatic load (when the stress can be overcome, “eustress”) to allostatic overload (when the stress cannot be overcome and becomes “distress”) (5, 11). The dominant hormone, cortisol or corticosterone, respectively, is pleiotropic and affects all major homeostatic systems of the vertebrate's body. Besides modulating actions, which alter an organism's response to a stressor, also preparative actions, which alter the organism's response to a subsequent stressor or aid in adapting to a chronic stressor, are distinguished (2). Hereby, a plethora of physiological processes are modulated including central nervous system (CNS) and cardiovascular functions, the metabolic system [e.g., bone metabolism (12), stimulation of gluconeogenesis, proteolytic processes in the muscle and lipolysis in the adipose tissues to increase plasma glucose levels)], the immune system (inflammatory response and lymphocyte production), growth, reproduction, and behavior (13). Furthermore, physiological amounts of glucocorticoids are also essential for normal renal tubular function and thus for water and electrolyte homeostasis (14, 15).

The perception of potential stressors by an individual varies (16, 17) and depends on various factors including but not limited to the species, genetic background, previous experiences (18), gender (19), age, and types as well as duration of the stressors (20, 21). The stress response will vary accordingly between individuals and physiological and behavioral responses tend to be associated in distinct suites of correlated traits, called “stress coping styles” (22). Hereby, the proactive stress coping style (active coping or “fight-flight”) is associated with low HPI or HPA axis responsiveness, but with high sympathetic reactivity, and is characterized by a high level of active avoidance, aggression and other actions indicating active attempts to counteract the stressful stimulus. The opposite is seen in reactive coping (passive coping or “conservation-withdrawal”) (22).

In all, the sheer diversity in potential stressors, individual perception and subsequent response to these stressors, and the plethora of metabolic processes mediated by glucocorticoids render accurate identification and quantification of the stressors experienced by an individual pivotal.

## Analysis of the dominant glucocorticoid is affected by other steroids

### By the less dominant glucocorticoid

The vertebrate stress response is mediated by the stress system which is activated when encountering environmental stressors but also when the body is at rest, hereby responding to various signals (e.g., circadian, neurosensory, blood-borne, and limbic) (23). The noradrenergic synthesizing neurons of the locus coeruleus/norepinephrine-central sympathetic system in the brainstem as well as the corticosteroid releasing hormone (CRH) and arginine vasopressin (AVP) synthesizing neurons of the hypothalamic paraventricular nuclei (PVN) comprise the central components, while the systemic sympathetic and adrenomedullary nervous systems and the HPI or HPA axis comprise the peripheral components of the stress system (24). Once triggered, CRH stimulates the release of adrenocorticotropic hormone (ACTH) from the pituitary, which results in glucocorticoid release, mainly cortisol, and corticosterone depending on the species, from the head kidney or adrenal gland, respectively. In ray-finned fish, cortisol predominates but corticosterone is also present; in the remaining fish species, the dominant or sole glucocorticoid varies. In this framework, 11-deoxycortisol in agnate fish (25); 1α-hydroxycorticosterone in sharks and rays (26); and 11-deoxycorticosterone in teleost fish (27, 28), were shown to be active glucocorticoids. In amphibians, reptiles and birds, the dominant glucocorticoid is corticosterone, while mammals, most placentals and marsupials secrete primarily cortisol. However, some rodents (e.g., rats and mice) secrete primarily or only corticosterone, whereas most other rodents secrete primarily or only cortisol (e.g., guinea pigs), while hamsters secrete both glucocorticoids in equal quantities. As a consequence, the less dominant glucocorticoid should be considered during analytical validation as it can cause cross-reactivity and subsequently bias glucocorticoid quantification.

### By other steroids

Glucocorticoids have a typical steroid structure consisting of a cyclopentaphenanthrene nucleus comprising three fused cyclohexane rings in a non-linear arrangement and a terminal cyclopentane ring. Most glucocorticoids possess a Δ4-3-keto group, a carbon ketol side-chain at C_17_ and generally an oxygen function at C_11_. The orientation of the groups attached to the steroid ring system is pivotal for the biological activity (29). As a consequence, other steroids including (i) androgens (C_19_-steroids such as testosterone); (ii) estrogens (C_18_-steroids such as estrone); (iii) mineralocorticoids (C_21_-steroids such as aldosterone); and (iv) progestagens (C_21_-steroids such as progesterone) (30), can be considered as physical-chemical similar molecules and should be taken into account during analytical validation as these compounds can cause cross-reactivity and subsequently bias glucocorticoid quantification.

### By direct precursors of the dominant glucocorticoid and the dominant glucocorticoid produced in “extra-interrenal” or “extra-adrenal” tissues

All steroids are derivatives of cholesterol (C_27_H_46_O) (31). Though, glucocorticoids were initially thought to be exclusively synthetized by the interrenal or adrenocortical cells, respectively, numerous studies have shown that they are also synthesized locally in so called “extra-interrenal” or “extra-adrenal” tissues (32). At present, these tissues include but are not limited to: primary lymphoid organs (33), intestine (34), CNS (35), cardiovascular system (36), skin (37–39), hair follicle (40), lung (41), kidney (42), and retina (43).

As a consequence, quantification of the dominant glucocorticoid produced by the HPI or HPA axis can be biased by direct precursors of the dominant hormone and the dominant hormone itself produced in extra-interrenal or extra-adrenal tissues, making the quantification (or at least analytical validation) of these other glucocorticoids of importance.

### By the manner how glucocorticoids are regulated

Systemically, glucocorticoid levels are influenced by distinct brain regions including structures of the limbic system (i.e., amygdala and hippocampus) and the midbrain (i.e., prefrontal cortex) (44) as well as by the hypothalamus, pituitary, and interrenal cells or adrenal cortex, respectively (45). In addition, the glucocorticoid pathway is controlled by the dominant glucocorticoid through a negative feedback loop. Besides this stress reactivity, glucocorticoid release is under control of a circadian clock (46). In humans the secretion of cortisol from the adrenal glands was shown to follow a diurnal cycle with a profound increase after awakening (47, 48).

Local regulation of glucocorticoid levels is mediated by access to target cells mediated by carrier proteins (49), by pre-receptor metabolism due to metabolic enzymes and by the availability of glucocorticoid (GR) and mineralocorticoid (MR) receptors.

#### By the non-free dominant glucocorticoid in the blood

Glucocorticoid levels vary rapidly due to the pulsatile nature of its secretion, rendering the dynamics of its binding critical determinants of tissue levels of free hormone and consequent hormone signaling. In most vertebrate species, the major proportion of circulating glucocorticoids are bound to a plasma glycoprotein called corticosteroid binding globulin (CBG) (50, 51). Subsequently, the free fraction is small (52). Since CBG is too large to leave the capillaries under normal circumstances, glucocorticoids bound to it remain in circulation. According to the “free hormone” hypothesis, it is the concentration of free, unbound hormone that determines how much glucocorticoids diffuses out of the capillaries and reaches the tissues. However, as CBG-bound glucocorticoids were shown to be released by enzymatic cleaving of the CBG molecule (53) and cell surface receptors for the CBG-glucocorticoid complex were shown to be present in certain tissues (54), one could argue that the glucocorticoid dissociation from CBG is part of the mechanism that makes the hormone biologically active.

In all, when focusing on cortisol producing vertebrates, cortisol is transported in blood more than 90% protein bound, approximately 70% with high affinity to CBG and 20% with low affinity to albumin, but it dissociates so rapidly that it is generally thought to be free (55). However, evidence indicates a dichotomous pattern with respect to CBG in these vertebrates: (i) a dominant branch where high levels of CBG bind most of the glucocorticoid which applies to the majority of vertebrates; and (ii) a smaller branch where low levels of CBG bind almost none of the glucocorticoid which applies to the fish (56). As a consequence, glucocorticoid analysis should be analytically validated to ensure that solely the free fraction of cortisol is quantified.

#### By phase I metabolites of the dominant glucocorticoid present in the body

Intracellular cortisol within the endoplasmatic reticulum of cells is regulated by local enzymes in a tissue-specific way independently of its plasma concentration (57). The intracellular enzyme 11β-hydroxysteroid dehydrogenase (11β-HSD) is bidirectional (58): 11β-HSD type 1 is a reductase that converts the 11-keto metabolite cortisone to its active form 11-hydroxy cortisol, amplifying glucocorticoid action in liver and visceral adipose tissue, but also in brain, bone, gonad, muscle and other GR-expressing tissues including the eye, while 11β-HSD type 2 catalyzes the oxidation of cortisol to cortisone (a hydroxyl group at C_11_ becomes a carboxyl group) and is co-expressed with the MR in the kidney, colon and salivary gland and inactivates cortisol to cortisone, thereby enabling aldosterone to bind to the MR (59, 60). In addition, cortisone was found to be further reduced to 20β-hydroxycortisone by 20β-HSD type 2 (61). As a consequence, glucocorticoid analysis should include cortisone as the latter is rapidly interconverted to and from cortisol as well as 20β-dihydrocortisone.

Corticosteroids affect a variety of target tissues over a broad range of time scales, ranging from slow gene transcription dependent to rapid gene transcription independent actions. Following uptake from the circulation, binding can occur by the two major functional groups of vertebrate corticosteroid receptors: GR and MR distinguished by their amino acid sequences and ligand specificity (62, 63). Most studies were performed on human intracellular genomic receptors [gGR reviewed by (64) and gMR reviewed by (65) as well as by (66)] regulating transcriptional activity of steroid target genes. Far less is known regarding the non-genomic effects mediated by the extracellular membrane glucocorticoid (mGR) and mineralocorticoid (mMR) receptors [for review see (67)], which allow rapid modulation of synaptic transmission and membrane ion currents hereby playing a key role in signal transduction at the synapse, the key neuron-to-neuron interface involved in learning and memory and as such in traumatic memories during times of stress (68, 69). As a consequence, glucocorticoid analysis should take into account the effect of phase I metabolites present in the body (i.e., cortisone and 20β-dihydrocortisone) as both compounds could potentially bind to GR and MR and are also excreted in minor proportions to the environment (see further).

### By phase I metabolites of the dominant glucocorticoid present in the environment

The dominant glucocorticoid, cortisol or corticosterone, respectively, is controlled by the ratio of *de novo* synthesis to catabolism by the action of the respective enzymes involved. In this framework, steroids undergo extensive bio-transformations which decrease their biological activity and increase their water solubility by converting them to hydrophilic compounds that can be excreted. In general, these bio-transformations are divided into: (i) phase I metabolism which usually includes oxidation (e.g., hydroxylation) and/or reduction (e.g., hydrogenation) reactions; and (ii) phase II metabolism which usually involves conjugation reactions with polar groups such as glucuronide or sulfate and resulting into a highly hydrophilic product, which facilitates excretion in the urine or feces.

Cortisol and cortisone are metabolized in the liver (70). The main pathways of phase I metabolic reaction include: (i) oxidation and reduction at C_11_; (ii) reduction of the C_4_-C_5_ double bond; and (iii) reduction at C_20_ (71, 30, 72). In a next step, (allo)-tetrahydrocortisol (THF) and (allo)-tetrahydrocortisone (THE) is (i) conjugated at a hydroxy group rapidly with glucuronic acid or sulfate and excreted in the urine or (ii) cleaved to the C_19_ steroids 11-hydroxy or 11-oxo-androsterone or etiocholanolone. In humans, non-metabolized cortisol and cortisone were shown to comprise only about 0.1% of the total urinary cortisol metabolites. At least 90% of the tetrahydro-derivatives of cortisol and cortisone are excreted into the urine as glucuronide or sulfate conjugates (73). Alternatively, reduction of the 20-oxo group by 20α- or 20β-hydroxysteroid dehydrogenase yields α and β cortols and cortolones, respectively, with subsequent oxidation at the C_21_ position to form the extremely polar metabolites, cortolic, and cortolonic acids (71). In addition, hydroxylation at C_6_ to form 6β-hydroxycortisol as well as reduction of the C_20_ position, which may occur without A ring reduction giving rise to 20α- and 20β-hydroxycortisol are described (74).

Overall, approximately 50% of secreted cortisol appears in the urine as THF/allo-THF/THE, 25% as cortols/cortolones, 10% as C_19_O_3_ steroids (androstanes), and 10% appears as cortolic/cortolonic acids. The remaining 5% metabolites are free, non-conjugated steroids (cortisol, cortisone and 6β- and 20α/20β-metabolites of cortisol and cortisone). As a consequence, glucocorticoid analysis should include the most abundant phase I metabolites such as THF and THE as they are indicative for possible contamination of the sample with glucocorticoids from urine, feces, water, as well as from anthropogenic contamination (e.g., from hands).

## Analysis of the dominant glucocorticoid is affected by the tissue used for glucocorticoid quantification

The type of tissue used for glucocorticoid quantification is of utmost importance as each tissue incorporates glucocorticoids in accordance with the processes by which it is formed hereby defining the timeframe of interrenal or adrenocortical activity that the tissue represents. Subsequently, a proper tissue for chronic stress quantification should allow a retrospective (i.e., over a certain period of time) view of the stress axis activity, and subsequently should possess the capacity to incorporate glucocorticoids in a stress (i.e., in reaction to stress full stimuli eliciting a glucocorticoid mediated response) and time (i.e., over a certain period of time) dependent manner (75). The type of tissue also determines the structural changes of the dominant glucocorticoid that may occur via processes of conjugation to glucuronides and sulfates, metabolic conversion via enzymatic action and bacterial breakdown (8). As a consequence, the effect of the tissue on the analysis results, as the latter can be enhanced or suppressed by tissue specific compounds, should be analytically validated. In practice, the choice of tissue depends on various factors including but not limited to: (i) the species; (ii) the nature of the study; (iii) acute vs. chronic stress quantification; (iv) the tissues available for sampling; and (v) logistical feasibility. Table 1 provides an overview of the temporal window of stress axis (re)activation that is being reported in tissues commonly used for glucocorticoid analysis across vertebrates. Hereby, it should be noticed that at present no tissue for chronic stress quantification exists for amphibians.

**Table 1:** Tissues commonly used for glucocorticoid analysis across vertebrates.

**Tissue**	**Temporal window on HPI/HPA (re)activity**	**References**
Vertebrate egg	Maternal deposition	(76)
Vertebrate plasma/serum	Snapshot	(57)
Whole body of fish larva	Snapshot	(77)
Mammalian saliva	Minutes	(78)
Vertebrate urine	Minutes to hours	(72)
Vertebrate feces	Minutes to days	(79)
Vertebrate excreta	Minutes to days	(80)
Water	Minutes to days	(81)
Reptilian shed skin	Weeks to months	(82)
Avian feather	Weeks to months	(83)
Fish scale	Weeks to years	(75)
Mammalian hair	Weeks to years	(84)

## Analysis of the dominant glucocorticoid is affected by the analytical methodology used

Glucocorticoids are measured using a wide variety of analytical methods including radio- (RIA) and enzyme (EIA) immunoassay, gas chromatography (GC), high performance liquid chromatography coupled to ultraviolet or fluorescence detection (HPLC-UV or FL), gas or liquid chromatography coupled to tandem mass spectrometry (GC- or LC-MS/MS) as well as sensor based techniques. In practice, the technique of choice depends mainly on the availability of qualified operators and sophisticated equipment in the laboratory.

### By screening methods

Immunoassays are most often chosen because they are fast, cheap, easy to perform, and commercially available for the dominant glucocorticoid in widely used tissues such as plasma of well-studied vertebrate species. RIA and EIA are both competitive binding assays necessitating an antibody directed against certain parts of the dominant glucocorticoid. While RIAs rely on a radioactive isotope (e.g., tritium or iodine) to generate a radioactive signal, EIAs use enzymes to generate a colorimetric signal to quantify the dominant glucocorticoid. Though immunoassays are sensitive (i.e., sufficient low levels can be detected) for the glucocorticoid of interest, major disadvantages are the lack of specificity (i.e., as they show high cross-reactivity with precursors and phase I metabolites of the targeted glucocorticoid as well as with substances with similar physical-chemical properties such as other steroids due to the poly-reactive nature of antibodies), the high lot-to-lot variation of antibodies (85), and the necessity to measure hormones individually. For example, Rettenbacher et al. (86) stated that their results for egg corticosterone could be explained by cross-reactions of the antibody used in the corticosterone EIA with other steroids, probably of gonadal origin as Hackl et al. (87) found a similar distribution pattern for progesterone. Subsequently, immunoassays should always be analytically validated in-depth.

The drawbacks of immunoassays have stimulated the development of new screening methods. Electrochemical biosensors have shown potential for fast, accurate and sensitive analysis of glucocorticoids. However, a continuing challenge is the sensitivity and stability of the surface bound bio-recognition molecules, which depends on the matrix used for their immobilization on the sensor (88). Besides the use of antibodies, molecular imprinting, which involves the synthesis of polymers in the presence of a template to produce the complementary binding sites with specific recognition ability, is also used. During this formation, the functional monomers are polymerized in the presence of a template, which is subsequently removed by washing and/or extraction after polymerization, resulting in a molecularly imprinted polymer (MIP) (89). A library of cortisol-imprinted polymers was prepared by Baggiani et al. (90), while Moreno-Guzmán et al. (91) reviewed the existing immunosensors for human cortisol.

In all, the lack of or insufficient in-depth analytical validation is the main cause of inconsistent results generated by immunoassays in the pertinent literature.

### By confirmation methods

For confirmatory purposes, chromatographic techniques such as GC and LC, especially when coupled to (tandem) MS, are preferred since they allow a high resolution as required for complex biological tissues (92). Major disadvantages are the need for qualified operators and sophisticated equipment, high costs and complex sample preparations.

Significant improvement in the specificity of glucocorticoid measurements was achieved with the introduction of GC-MS/MS, however, accurate quantification is limited to analytes which can be derivatized (93) in order to increase their volatility (94). Because of limited sensitivity, low throughput and labor-intensive sample preparation, GC-MS/MS is not optimal for measuring glucocorticoid profiles. HPLC is well suited for the separation of glucocorticoids, though when coupled to UV or FL it lacks the sensitivity and specificity to distinguish glucocorticoid traces from the biological matrix background (29). Because of its inherent sensitivity and selectivity, LC-MS/MS is considered the gold standard method for quantitation of glucocorticoids in complex biological tissues (95, 96, 92). It has the further advantage of having the capability to perform multi-compound assays, even across compound classes (97).

## Analysis of the dominant glucocorticoid is affected by the lack of analytical validation

Overall, glucocorticoid levels to be quantified are considered “trace levels” as they are situated in the ppb (μg kg^−1^ or μg L^−1^) and ppt (ng kg^−1^ or ng L^−1^) range. Regardless the sample tissue and analytical methodology used, it is pivotal to demonstrate that results are accurate, precise, and not biased by interfering compounds rendering results highly reliable. Subsequently, every procedure [i.e., parameter(s)/tissue combination using a specific analytical methodology] should be analytically validated. In this framework, working according the criteria of international standards such as EN ISO/IEC 17025 (98) and Commission Decision No. 2002/657/EC (99, 100), whereby experiments are carried out by well trained and authorized personnel in a controlled environment are a must. Hereby, the use of calibrated equipment, products with a certificate of analysis as well as performing all tests in standardized conditions hereby registering all details in logbooks is of importance. In addition, determination of the performance characteristics such as accuracy, trueness, precision, sensitivity, specificity and cross-reactivity with structurally related compounds are of utmost importance as they can influence the interpretation of results between studies. In particular immunoassays are prone to be biased by this as the used antiserum differs between assays leading to differences in cross-reactivity (8). Subsequent, physiological (i.e., by pharmacologically induced physiological changes in circulating glucocorticoid levels and to evaluate whether these changes are reflected in measured concentrations afterwards) as well as biological (i.e., glucocorticoid measurements in relation to cortical activity and the experience of stress) validation is needed in order to state that the method is fit for purpose (7).

As a consequence, one should try to use methods developed in an EN ISO/IEC 17025 regulated environment and analytically validated according the criteria of international standards as this ensures full traceability and quality of the results in time.

## Conclusion

At present, most studies in the pertinent literature have focused on the quantification of the dominant glucocorticoid, cortisol or corticosterone depending on the species, using immunoassays. Hereby, one should bare in mind that: (i) results are prone to bias by cross-reactivity from other glucocorticoids as well as substances with similar physical-chemical properties, making analytical validation a must; (ii) immunoassays are screening methods which do not allow quantification of multiple substances, making them not suited for quantification of a glucocorticoid profile needed to obtain a more accurate and complete view on the HPI or HPA axis (re)activity, respectively. However, in-depth validated immunoassays for the dominant glucocorticoid can be useful in cases when only an indication (i.e., qualitative) of stress is needed. In addition, the use of pooled samples (e.g., for whole body of fish larva) renders it impossible to take into account the coping style of a single individual.

As a consequence, internationally validated confirmation methods for quantification of a glucocorticoid profile comprising: (i) the dominant hormone (e.g., cortisol); (ii) its direct precursors (i.e., 17α-hydroxyprogesterone and 11-deoxycortisol; as both will certainly lead to cortisol production); (iii) its endogenously present phase I metabolites (i.e., cortisone and 20β-dihydrocortisone; as feedback regulation of cortisol at pre-receptor level is mediated by 11β-HSD and 20β-reductase, respectively); and (iv) the most abundant more polar excreted phase I metabolites (i.e., tetrahydrocortisol and tetrahydrocortisone; to establish if exogenous glucocorticoids present in the environment (e.g., from water) or anthropogenic derived glucocorticoids (e.g., from hands) may have influenced the results) in non-pooled samples are pivotal in stress research across vertebrates.

## Author contributions

The author confirms being the sole contributor of this work and has approved it for publication.

### Conflict of interest statement

The author declares that the research was conducted in the absence of any commercial or financial relationships that could be construed as a potential conflict of interest.
